# ﻿Evolutionary dynamics of the B chromosomes in the fish species *Prochiloduslineatus* Valenciennes, 1837 ﻿of the Paraná River Basin

**DOI:** 10.3897/compcytogen.19.135127

**Published:** 2025-01-20

**Authors:** Manolo Penitente, Caio Augusto Gomes Goes, Rodrigo Zeni dos Santos, Ricardo Utsunomia, Fausto Foresti, Fabio Porto-Foresti

**Affiliations:** 1 Universidade do Estado de Mato Grosso (UNEMAT), Rua Rui Barbosa, Diamantino, Mato Grosso, Brazil Universidade do Estado de Mato Grosso (UNEMAT) Diamantino Brazil; 2 Faculdade de Ciências, Universidade Estadual Paulista (UNESP), Avenida Edmundo Carrijo Coube, Bauru, SP, Brazil Universidade Estadual Paulista (UNESP) Bauru Brazil; 3 Departamento de Morfologia, Instituto de Biociências, Universidade Estadual Paulista - UNESP, Distrito de Rubião Junior, 18618-970, Botucatu, SP, Brazil Universidade Estadual Paulista - UNESP Botucatu Brazil

**Keywords:** Cytogenetics, migratory fish, Neotropical fishes, supernumerary chromosomes

## Abstract

The fish species *Prochiloduslineatus* has an interesting B chromosome system, with three morphological types as acrocentric, metacentric, and submetacentric. However, most cytogenetic studies on this species are restricted to the natural population of the Mogi Guaçu River. Given this, the present work aimed to study the structure karyotypic profile as well as the occurrence of supernumeraries in *P.lineatus* in several localities in the Paraná River basin, where this species is abundant. The results obtained showed a predominantly conserved karyotypic macrostructure and the presence of B chromosomes in all the seven localities studied, with the exception of the Apa River. Additionally, new variants of morphological characteristics were found in the population of the Batalha River (Reginópolis). These results allow us to infer that there is a large occurrence of B chromosomes in this species, with important differences in B chromosome frequency between the populations, especially in acrocentric and submetacentric B variants. Considering the possible origin and evolution of B chromosomes in *P.lineatus*, our results allow us to describe the dispersion of metacentric B variants, in contrast with the elimination observed in acrocentric and submetacentric variants.

## ﻿Introduction

The ichthyofauna of the Neotropical region is one of the richest in the world, with approximately 9100 species (Reis et al. 2016). One of the main orders of Neotropical fishes is Characiformes, which includes the family Prochilodontidae, comprising 21 species and divided into three genera: *Ichthyoelephas* Posada, 1909 with two species; *Prochilodus* Agassiz, 1829 with 13 species; and *Semaprochilodus* Fowler, 1941 with six species (Fricke et al. 2024; Froese and Pauly 2024). *Prochilodus* is the most abundant among these genera and is composed of detritivore fish of moderate size. In this context, *Prochiloduslineatus* Valenciennes, 1837 is characterized by its migratory behavior, with the shoals traveling hundreds of kilometers in the breeding season (Melo et al. 2017). *P.lineatus* populations usually exhibit low genetic variation (Garcez et al. 2011; Rueda et al. 2013), as also observed in other species of the Prochilodontidae (Machado et al. 2017; Landínez-García et al. 2020). In a similar way, cytogenetic studies have been demonstrating a diploid chromosome number (2n = 54) and monotonous karyotype structure with only metacentric and submetacentric pairs in *Prochilodus* (Pauls and Bertollo 1983, 1990; Voltolin et al. 2013), with the remarkable presence of B chromosomes in species such as *Prochilodusargenteus* Spix et Agassiz, 1829 (Penitente et al. 2015), *Prochiloduscostatus* Valenciennes, 1850 (Melo et al. 2017), *Prochilodusbrevis* Steindachner, 1875 (Pauls and Bertollo 1990), *Prochilodusmariae* Eigenmann, 1922 (Oliveira et al. 2003), and *P.lineatus* (Pauls and Bertollo 1983; Penitente et al. 2016; Stornioli et al. 2021). B chromosomes are selfish supernumerary elements that typically do not follow Mendelian inheritance laws (Camacho et al. 2000) and are found in the genomes of various plants, fungal species, and animal species (Camacho et al. 2000; Douglas and Birchler 2017). These elements are usually heterochromatic, but next-generation sequencing is demonstrating that their structure comprises fragments of A chromosomes (Ruban et al. 2017; Oliveira et al. 2024). In addition, recent transcriptomic analyses are registering the expression of these elements, including protein-encoding (Ruban et al. 2017; Oliveira et al. 2024). The origin of B chromosomes could be intraspecific, with these elements arising from A chromosomes of their host species (Mestriner et al. 2000; Jesus et al. 2003; Artoni et al. 2006), or interspecific, because of hybridization events (Schartl et al. 1995; Tosta et al. 2014), and repetitive elements can provide evidence of the origin mechanisms of these elements. In *P.lineatus*, it is assumed that the B chromosomes have an intraspecific origin due to the sharing of a satellite DNA (PliSat05-178) only between the B chromosomes and the telomeric region of the pair 4 (Stornioli et al. 2021).

Despite the similar content of repetitive sequences (Stornioli et al. 2021), *P.lineatus* presents three different variants of B chromosomes: acrocentric (small-sized), metacentric (medium-sized), and submetacentric (large-sized) (Artoni et al. 2006; Penitente et al. 2013). A large variation in the number of B chromosomes in an individual is documented, with individuals containing 0 to 9 B chromosomes (Voltolin et al. 2011). In addition, repetitive sequences are shared by *P.lineatus*, *P.costatus*, and *P.argenteus* B chromosomes, indicating a common origin of B chromosomes in *Prochilodus* (Melo et al. 2017).

Despite the widespread distribution of *P.lineatus* in South American watersheds, the majority of knowledge on the structure, inheritance, and evolution of this species’ B chromosomes is limited to the Mogi-Guaçu River and Sapucaí River (Voltolin et al. 2011; Penitente et al. 2013, 2015, 2016; Stornioli et al. 2021), with few information available from other locations. The current study aimed to address this knowledge gap in other locations while also updating the B chromosome studies in *P.lineatus* with new data and statistical analyses on its quantity, presence, and morphology in seven sites within the Paraná River basin.

## ﻿Materials and methods

**Figure 1.**
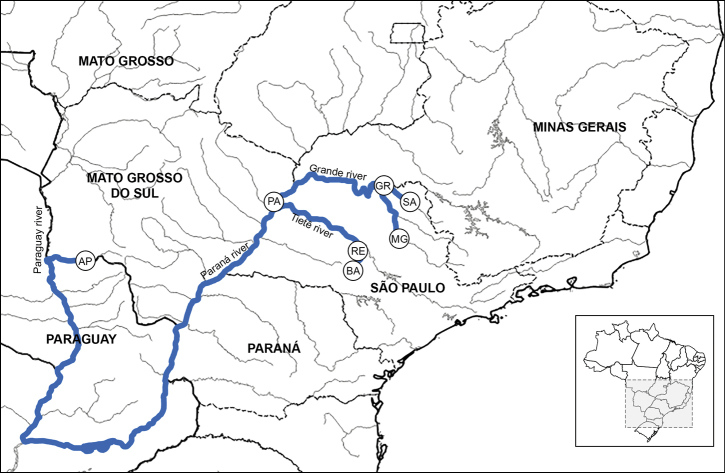
Collection sites of *P.lineatus* at Paraná River Basin.

We collected 148 individuals of *P.lineatus* in seven localities of the Paraná River basin (Table 1, Fig. 1). All samples were obtained with authorization from the relevant organizations (MMA/IBAMA/ICMBio/SISBIO-18884-1). Cytogenetic preparations were obtained through lymphocyte culture technique, as established by Fenocchio and Bertollo (1988). Nucleolus organization regions (NORs) are shown following the protocols established by Howell and Black (1980), and heterochromatic regions were visualised using the C-banding, as established by Sumner (1972). All images were captured using the software CellSens Standard 1.14 (Olympus) with a digital camera (Olympus Qcolor5) on a fluorescence microscope (BX50, Olympus).

**Table 1.** Number and geographic origin of *P.lineatus* specimens collected at each point of the Paraná River Basin.

Population	Code	N	Geographic coordinates
Mogi Guaçu	MG	32	-21.9267528, -47.3669111
Sapucaí	SA	23	-20.5724083, -47.7827416
Grande	GR	20	-20.1258167, -48.5738056
Paraná	PA	30	-20.7767028, -51.6404721
Reginópolis	RE	15	-21.8441917, -49.2194721
Bauru	BA	8	-22.3820556, -49.1153610
Apa	AP	20	-22.2497111, -56.9268693

Frequency comparisons of each B chromosome variant among populations were made using one-way ANOVA with the BioEstat 5.3 software (Ayres et al. 2007). Only values with P less than 0.05 were considered statistically significant (P < 0.05).

## ﻿Results

**Figure 2.**
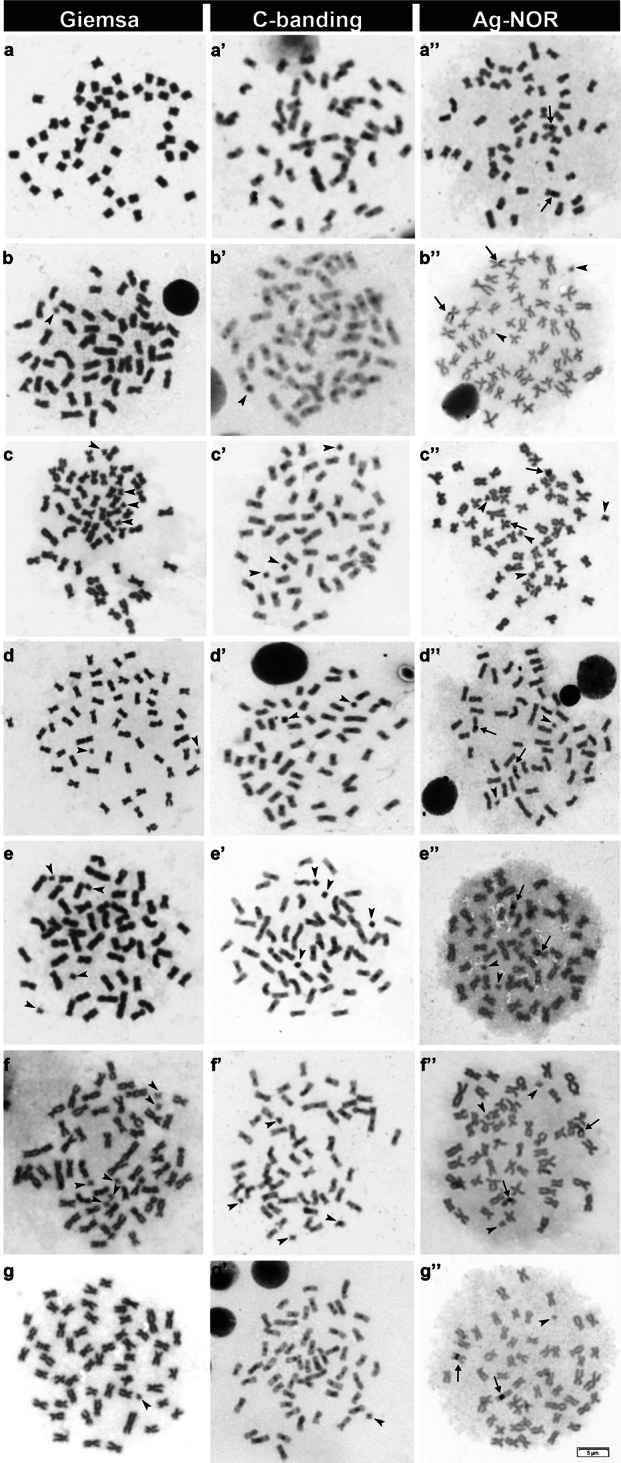
Metaphases of all analyzed populations of *P.lineatus* stained totally (Giemsa) or differentially (C bands, Ag-NORs). The locations are Apa (**a**), Mogi Guaçu (**b**), Sapucaí (**c**), Batalha – Reginópolis (**d**), Grande (**e**), Batalha – Bauru (**f**), and Paraná (**g**). Arrowheads indicate B chromosomes, and arrows indicate NOR regions.

All the individuals collected in this work had 2n = 54, with only meta- or submetacentric chromosomes and a fundamental number of 108 (Fig. 2). The C-banding demonstrates the presence of heterochromatin only in the centromeric portion of all chromosomes, without differences between populations (Fig. 2). In addition, all B chromosomes observed in the specimens were completely heterochromatic (Fig. 2). The Ag-NOR technique also demonstrated conservation in the karyotype of *P.lineatus* in all populations analyzed, with all individuals demonstrating just one block in the long arm of one pair of submetacentric chromosomes (Fig. 2). None of the B chromosomes had Ag-NOR signals.

**Figure 3.**
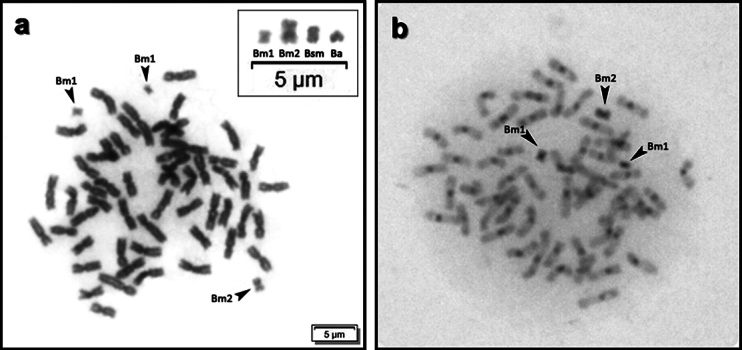
Metaphases with B chromosome variants of the species *Prochiloduslineatus* (Bm1 = metacentric, Ba = acrocentric, Bsm = submetacentric) and the discovery of a new metacentric macrochromosome (Bm2), present in the RE population **a** giemsa staining **b** c banding showing this variant completely heterochromatic.

The populations of *P.lineatus* analyzed here have demonstrated heterogeneity related to the presence of B chromosomes (Table 2). The Apa River population (APA) was the only one without the presence of any B chromosome in all the individuals analyzed (Table 2). The three morphologies of B chromosomes ever described were found in the locations Mogi Guaçu (MG), Sapucaí (SA), and Grande (GR), representing the Rio Grande basin. The Paraná River (PA) demonstrated the prevalence of metacentric B chromosomes, with only one individual having an acrocentric B chromosome. In two points of the Rio Batalha (RE and BA), representing the Tietê River basin, only metacentric B chromosomes were found. However, in RE, one individual presented a metacentric macrochromosome B, representing a new variation of this element in *P.lineatus* (Fig. 3), also completely heterochromatic.

**Table 2.** Statistical analyses of the types of B found in *P.lineatus* in the seven sampled points. (M – metacentric, SM – submetacentric, A - acrocentric). SD = Standard deviation.

B variant		MG	SA	GR	PA	RE	BA	AP
M	Mean	1.8750	2.0435	2.3000	1.2000	2.0667	3.0000	0.0000
	SD	0.1665	0.1831	0.2524	0.1390	0.2063	0.6547	0.0000
S	Mean	0.5000	0.2609	0.4500	0.0000	0.0000	0.0000	0.0000
	SD	0.1004	0.1128	0.1535	0.0000	0.0000	0.0000	0.0000
A	Mean	0.0625	0.3478	0.6000	0.0333	0.0000	0.0000	0.0000
	SD	0.0435	0.1194	0.1522	0.0333	0.0000	0.0000	0.0000
Total	Mean	2.4375	2.6364	3.3500	2.4300	2.0667	3.0000	0.0000
	SD	1.2684	1.0272	1.5652	0.7279	0.7988	1.8516	0.0000

**Figure 4.**
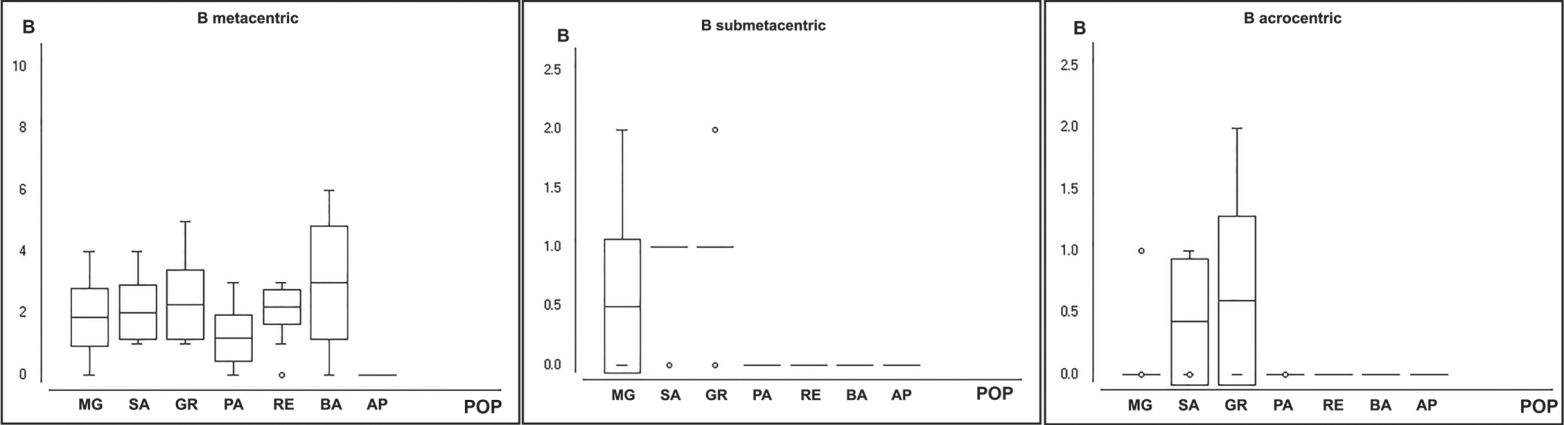
Frequency of metacentric, submetacentric, and acrocentric variants of B chromosomes in locations, respectively.

The statistical analyses using one-way ANOVA on the frequencies of different types of B chromosomes demonstrated differentiation among localities, with F = 18.1307, P < 0.0001 for metacentric B chromosome, with degrees of freedom (df) = 6, F = 6.5932, P < 0.0001 for submetacentric B chromosome (df = 6), and F = 8.1826, P < 0.0001 for acrocentric B chromosome (df = 6) (Fig. 4).

## ﻿Discussion

Pioneering studies conducted by Pauls and Bertollo (1983, 1990) on the cytogenetics of fish of the genus *Prochilodus* Agassiz, 1829 suggested the existence of homogeneity of basic karyotypic characters. Several studies have shown that different species of *Prochilodus* analyzed cytogenetically presented a diploid number composed of metacentric and submetacentric chromosomes, with a fundamental number equal to 108 (Pauls and Bertollo 1983; 1990; Oliveira et al. 1997; Cavallaro et al. 2000; Jesus and Moreira-Filho 2003; Voltolin et al. 2009, 2013). Our data corroborates this affirmation, with all individuals analyzed in this work demonstrating the same pattern of NOR, heterochromatin regions, and fundamental number (Fig. 2).

Although diploid number and basic cytogenetic markers are conserved, B chromosomes follow a directly opposite path. We found a large variation related to the frequency of B chromosomes in the analyzed populations, especially in the acrocentric and submetacentric variations, which could mean a population structure in *P.lineatus*. However, several studies related to the population dynamics of *P.lineatus* demonstrated a panmictic population for this fish (Garcez et al. 2011; Rueda et al. 2013). Behavior studies indicate that *P.lineatus* can migrate more than 1000 km, but this distance is significantly smaller to reproductive goals, varying between 100 and 400 km (Godoy 1975). So, the B chromosomes can demonstrate different meiotic drives in each locality observed in this work. Despite the differences in species, B chromosome systems, and the environment, different B chromosome frequencies are also observed in a population of *Psalidodonparanae* Eigenmann 1914. The animals of the Cascatinha stream (Botucatu, São Paulo, Brazil -22°53'22.5"S, 48°29'22.4"W), collected in different stretch of the river, demonstrated an accentuated difference in B chromosome frequency, varying from 65.7% of B chromosome carriers in stretch 1, next to the source of the river, in contrast to 10% of B chromosome carriers in the other two stretches of the river along (Porto-Foresti et al. 1997).

Intensive B chromosome analysis in *Prochilodus* has focused on its structure, origin, and inheritance (Voltolin et al. 2011; Penitente et al. 2013, 2016; Stornioli et al. 2021). The FISH mapping of satellite DNAs points to a common origin of these chromosomes in the genus *Prochilodus*, before the speciation of at least three carrier species (*P.lineatus*, *P.costatus* and *P.argenteus*). In addition, all variants of the B chromosomes of *P.lineatus* have the same repetitive composition and are probably derived from the autosomal pair 4 (Stornioli et al. 2021). This fact reinforces the hypothesis that there is a single origin to the variations of B in *P.lineatus*, from an ancestral acrocentric and the formation of an isochromosome, resulting in the actual metacentric. The other variants would be derived from the metacentric (Penitente et al. 2016), as the new metacentric macrochromosome B described in the Reginópolis population (present work). So, the possibility of a new event of origin that could modify the frequencies of Bs in the analyzed populations seems to be very improbable.

B chromosomes are considered selfish parasitic elements (Camacho et al. 2000; Douglas and Birchler 2017), and their inheritance mechanisms are a big question in carrier species. In grasshoppers, an elimination process during spermiogenesis is documented in the form of nonfunctional microspermatids (Cabrero et al. 2017). In *P.paranae*, females with B chromosomes have an inheritance pattern lower than B carrier males, suggesting an elimination process of Bs by the elimination of polar bodies (Goes et al. 2021). In *P.lineatus*, the variants have different transmission rates, with the metacentric being the only variation with inheritance rates above the Mendelian expectations (kb = 0.587), in contrast to the acrocentric and submetacentric variants (kb = 0.333 and 0.385, respectively), characterized by an elimination of B chromosomes process, following the B chromosome evolution cycle (Camacho et al. 2000). This data corroborates the data of this work, with the metacentric variation dispersed by almost all populations analyzed and the other two variants being very rare, restricted to some individuals of three populations. So, our hypothesis is that the acrocentric and submetacentric variants have already been lost in PA, RE, BA, and AP populations, persisting only in populations of MG, SA, and GR. This process seems to be more accentuated in submetacentric variation, with expressive presence only in the MG population. In contrast, the metacentric variant persists in all populations, with the exception of AP, due to the accumulation inheritance pattern of the metacentric.

Only the AP population shows individuals without B chromosomes (Stornioli et al. 2021). This population was isolated by the Sete Quedas waterfall, which was destroyed by the construction of the Itaipu hydroelectric plant. So, the absence of Bs in these animals can be explained by two hypotheses: 1) Bs were eliminated in this population and can appear again due to contact with animals from other populations; 2) Bs never existed in the AP population and now can appear for the first time due to contact with other populations. We consider hypothesis two invalid due to the origin of Bs anterior of the diversification of *P.lineatus* and two other species (Melo et al. 2017), showing the very old origin of these elements.

Several studies have been dedicated to the B chromosomes of *P.lineatus*, generating insights about their origin (Stornioli et al. 2021), repetitive sequence content (Jesus et al. 2003; Melo et al. 2017; Stornioli et al. 2021), and inheritance (Penitente et al. 2015). The present work reveals the evolutionary dynamic along the Parana River basin, revealing the manutention of metacentric variants and potential disappearance of the acrocentric and submetacentric variants. The perspectives about the studies of B chromosomes of *P.lineatus* include the genome assembling at chromosome level, which can potentially demonstrate genomic differences between the three variants and bring new light about the evolution of supernumeraries in *P.lineatus*.

## ﻿Conclusion

Our work presents an extensive panorama of B chromosome frequency in the *P.lineatus* population of the Alto Paraná Basins, corroborating theories about the evolution of these elements. We could verify the dispersion of metacentric variation, agreeing with the accumulation inheritance pattern, in contrast with the elimination process observed in acrocentric and submetacentric B chromosomes.

## ﻿Author contributions

Manolo Penitente - conceptualization, methodology, formal analysis, writing of original draft; Caio Augusto Gomes Goes - validation, formal analysis, data Curation, writing of original draft; Rodrigo Zeni dos Santos - formal analysis, data curation, writing of original draft; Ricardo Utsunomia - validation, formal analysis, data curation, writing of original draft, writing of review; Fausto Foresti - validation, writing of review and editing, visualization; Fabio Porto-Foresti - conceptualization, validation, resources, writing of review and editing, visualization, supervision, project administration, funding acquisition.

## Supplementary materials

Supplementary material 1 Number of B chromosome in *P.lineatus* of Mogi Guaçu population Data type docx https://binary.pensoft.net/file/1224173This dataset is made available under the Open Database License (http://opendatacommons.org/licenses/odbl/1.0/). The Open Database License (ODbL) is a license agreement intended to allow users to freely share, modify, and use this Dataset while maintaining this same freedom for others, provided that the original source and author(s) are credited.Manolo Penitente, Caio Augusto Gomes Goes, Rodrigo Zeni dos Santos, Ricardo Utsunomia, Fausto Foresti

## References

[B1] Artoni RF Vicari MR Endler AL Cavallaro ZI Jesus CM Almeida MC Moreira-Filho O Bertollo LAC (2006) Banding pattern of A and B chromosomes of *Prochiloduslineatus* (Characiformes, Prochilodontidae), with comments on B chromosomes evolution. Genetica 127(1): 277–84. 10.1007/s10709-005-4846-1 16850231

[B2] Ayres M Ayres JRM Ayres DL Santos AS (2007) BioEstat 5.0-Aplicações Estatísticas Nas Áreas Das Ciências Biológicas e Médicas: Sociedade Civil Mamirauá, 364 pp.

[B3] Cabrero J Martín-Peciña M Ruiz-Ruano FJ Gómez R Camacho JPM (2017) Post-meiotic B chromosome expulsion, during spermiogenesis, in two grasshopper species. Chromosoma 126(5): 633–44. 10.1007/s00412-017-0627-8 28190081

[B4] Camacho JPM Sharbel TF Beukeboom LW (2000) B-chromosome evolution. Philosophical Transactions of the Royal Society of London B: Biological Sciences 355(1394): 163–78. 10.1098/rstb.2000.0556 10724453 PMC1692730

[B5] Cavallaro ZI Bertollo LAC Perfectti F Camacho JPM (2000) Frequency increase and mitotic stabilization of a B chromosome in the fish *Prochiloduslineatus*. Chromosome Research 8(7): 627–34. 10.1023/A:1009242209375 11117359

[B6] Douglas RN Birchler JA (2017) B chromosomes. In: Bhat TA Wani AA (Eds) Chromosome structure and aberrations.Springer India, New Delhi, 13–39. 10.1007/978-81-322-3673-3_2

[B7] Fenocchio AS Bertollo LAC (1988) A simple method for fresh-water fish lymphocyte culture. Rev. Bras. Genét, 847–52.

[B8] Fricke R Eschmeyer WN Van der Laan R (2024) Eschmeyer’s Catalog of Fishes: Genera, Species, References. [acessed 17. September 2024]

[B9] Froese R Pauly D (2024) FishBase. http://www.fishbase.org/ [acessed 17. September 2024]

[B10] Garcez R Calcagnotto D Almeida‐Toledo LF (2011) Population structure of the migratory fish *Prochiloduslineatus* (Characiformes) from Rio Grande basin (Brazil), an area fragmented by dams. Aquatic Conservation: Marine and Freshwater Ecosystems 21(3): 268–75. 10.1002/aqc.1176

[B11] Godoy MP (1975) Peixes do Brasil, subordem Characoidei, bacia do Rio Mogi Guassu. Editora Franciscana, 846 pp.

[B12] Goes CAG Silva DMZA Utsunomia R Nascimento NF Yasui GS Senhorini JA Hashimoto DT Artoni RF Foresti F Porto-Foresti F (2021) Sex-dependent inheritance of B chromosomes in *Psalidodonparanae* (Teleostei, Characiformes) revealed by directed crossings. Zebrafish 18(6): 363–68. 10.1089/zeb.2021.0053 34935496

[B13] Howell WM Black DA (1980) Controlled silver-staining of nucleolus organizer regions with a protective colloidal developer: a 1-step method. Experientia 36(8): 1014–15. 10.1007/BF01953855 6160049

[B14] Jesus CM Galetti PMJ Valentini SR Moreira-Filho O (2003) Molecular characterization and chromosomal localization of two families of satellite DNA in *Prochiloduslineatus* (Pisces, Prochilodontidae), a species with B chromosomes. Genetica 118(1): 25–32. 10.1023/A:1022986816648 12737143

[B15] Jesus CM Moreira-Filho O (2003) Chromosomal location of 5S and 18S rRNA genes in *Prochiloduslineatus* (Characiformes, Prochilodontidae). Caryologia 56(3): 281–87. 10.1080/00087114.2003.10589336

[B16] Landínez-García RM Narváez JC Márquez EJ (2020) Population genetics of the freshwater fish *Prochilodusmagdalenae* (Characiformes: Prochilodontidae), using species-specific microsatellite loci. PeerJ 8:e10327. 10.7717/peerj.10327 PMC7666565 33240645

[B17] Machado VN Willis SC Teixeira AS Hrbek T Farias IP (2017) Population genetic structure of the Amazonian black flannelmouth characin (Characiformes, Prochilodontidae: *Prochilodusnigricans* Spix & Agassiz, 1829): contemporary and historical gene flow of a migratory and abundant fishery species. Environmental Biology of Fishes 100: 1–16. 10.1007/s10641-016-0547-0

[B18] Melo BF Sidlauskas BL Van der Sleen P Albert JS (2017) Prochilodontidae-Flannel Mouth Characiforms. In: Van der Sleen P Albert JS (Eds) Field guide to the fishes of the Amazon, Orinoco, and Guianas.Princeton, New Jersey: Princeton University Press, 170–171. 10.2307/j.ctt1qv5r0f

[B19] Melo S Utsunomia R Penitente M Sobrinho-Scudeler PE Porto-Foresti F Oliveira C Foresti F Dergam JA (2017) B chromosome dynamics in *Prochiloduscostatus* (Teleostei, Characiformes) and comparisons with supernumerary chromosome system in other *Prochilodus* species. Comparative Cytogenetics 11(2): 393–403. 10.3897/compcytogen.v11i2.12784 28919971 PMC5596993

[B20] Mestriner CA Galetti PMJ Valentini SR Ruiz IRG Abel LDS Moreira-Filho O Camacho JPM (2000) Structural and functional evidence that a B chromosome in the characid fish *Astyanaxscabripinnis* is an isochromosome. Heredity 85(1): 1–9. 10.1046/j.1365-2540.2000.00702.x 10971685

[B21] Oliveira JIN Cabral-de-Mello DC Valente GT Martins C (2024) Transcribing the enigma: the B chromosome as a territory of uncharted RNAs. Genetics 227(1): iyae026. 10.1093/genetics/iyae026 38513121

[B22] Oliveira C Nirchio M Granado A Levy S (2003) Karyotypic characterization of *Prochilodusmariae*, *Semaprochiloduskneri* and *S.laticeps* (Teleostei: Prochilodontidae) from Caicara Del Orinoco, Venezuela. Neotropical Ichthyology 1(1): 47–52. 10.1590/S1679-62252003000100005

[B23] Oliveira C Saboya SMR Foresti F Senhorini JA Bernardino G (1997) Increased B chromosome frequency and absence of drive in the fish *Prochiloduslineatus*. Heredity 79(5): 473–76. 10.1038/hdy.1997.186

[B24] Pauls E Bertollo LAC (1983) Evidence for a system of supernumerary chromosomes in *Prochilodusscrofa* Steindachner, 1881 (Pisces, Prochilodontidae). Caryologia 36(4): 307–14. 10.1080/00087114.1983.10797671

[B25] Pauls E Bertollo LAC (1990) Distribution of a supernumerary chromosome system and aspects of karyotypic evolution in the genus *Prochilodus* (Pisces, Prochilodontidae). Genetica 81(2): 117–123. 10.1007/BF00226450

[B26] Penitente M Daniel SN Senhorini JA Foresti F Porto-Foresti F (2015) Transmission behavior of B chromosomes in *Prochiloduslineatus* (Characiformes, Prochilodontidae). Cytogenetic and Genome Research 147(2–3): 179–85. 10.1159/000443384 26795613

[B27] Penitente M Daniel SN Scudeler PES Foresti F Porto-Foresti F (2016) B chromosome variants in *Prochiloduslineatus* (Characiformes, Prochilodontidae) analyzed by microdissection and chromosome painting techniques. Caryologia 69(2): 181–86. 10.1080/00087114.2016.1152113

[B28] Penitente M Foresti F Porto-Foresti F (2015) B chromosomes in the species *Prochilodusargenteus* (Characiformes, Prochilodontidae): morphological identity and dispersion. Comparative Cytogenetics 9(1): 79–87. 10.3897/CompCytogen.v9i1.8587 25893076 PMC4387382

[B29] Penitente M Voltolin TA Senhorini JA Bortolozzi J Foresti F Porto-Foresti F (2013) Transmission rate variation among three B chromosome variants in the fish *Prochiloduslineatus* (Characiformes, Prochilodontidae). Anais Da Academia Brasileira de Ciencias 85(4): 1371–1377. 10.1590/0001-3765201387611 24141415

[B30] Porto-Foresti F Oliveira C Maistro EL Foresti F (1997) Estimated frequency of B-chromosomes and population density of *Astyanaxscabripinnisparanae* in a small stream. Brazilian Journal of Genetics 20(3): 377–80. 10.1590/S0100-84551997000300004

[B31] Reis RE Albert JS Dario F Mincarone MM Petry P Rocha LA (2016) Fish biodiversity and conservation in South America. Journal of Fish Biology 89(1): 12–47. 10.1111/jfb.13016 27312713

[B32] Ruban A Schmutzer T Scholz U Houben A (2017) How next-generation sequencing has aided our understanding of the sequence composition and origin of B chromosomes. Genes 8(11): 294. 10.3390/genes8110294 29068386 PMC5704207

[B33] Rueda EC Carriquiriborde P Monzón AM Somoza GM Ortí G (2013) Seasonal variation in genetic population structure of Sábalo (*Prochiloduslineatus*) in the lower Uruguay River. Genetica 141(7): 401–407. 10.1007/s10709-013-9739-0 24068426

[B34] Schartl M Nanda I Schlupp I Wilde B Epplen JT Schmid M Parzefall J (1995) Incorporation of subgenomic amounts of DNA as compensation for mutational load in a gynogenetic fish. Nature 373(6509): 68–71. 10.1038/373068a0

[B35] Stornioli JHF Goes CAG Calegari RM dos Santos RZ Giglio LM Foresti F Oliveira C Penitente M Porto-Foresti F Utsunomia R (2021) The B chromosomes of *Prochiloduslineatus* (Teleostei, Characiformes) are highly enriched in satellite DNAs. Cells 10(6): 1527. 10.3390/cells10061527 34204462 PMC8235050

[B36] Sumner AT (1972) A simple technique for demonstrating centromeric heterochromatin. Experimental Cell Research 75(1): 304–306. 10.1016/0014-4827(72)90558-7 4117921

[B37] Tosta VC Marthe JB Tavares MG Fernandes-Salomão TM Pompolo SG Recco-Pimentel SM Perfectti F Campos LAO Camacho JPM (2014) Possible introgression of B chromosomes between bee species (genus *Partamona*). Cytogenetic and Genome Research 144(3): 220–226. 10.1159/000370171 25612643

[B38] Voltolin TA Penitente M Mendonça BB Senhorini JA Foresti F Porto-Foresti F (2013) Karyotypic conservatism in five species of *Prochilodus* (Characiformes, Prochilodontidae) disclosed by cytogenetic markers. Genetics and Molecular Biology 36(3): 347–352. 10.1590/S1415-47572013000300008 24130441 PMC3795166

[B39] Voltolin TA Senhorini JA Oliveira C Foresti F Bortolozzi J Porto-Foresti F (2009) Cytogenetic markers in wild population of Curimbatá (*Prochiloduslineatus*) from Mogi-Guaçu River. Cytologia 74(3): 281–287. 10.1508/cytologia.74.281

[B40] Voltolin TA Senhorini JA Foresti F Bortolozzi J Porto-Foresti F (2011) Intraspecific crosses resulting in the first occurrence of eight and nine B chromosomes in *Prochiloduslineatus* (Characiformes, Prochilodontidae). Genetics and Molecular Biology 34(2): 220–224. 10.1590/S1415-47572011005000009 21734820 PMC3115313

