# Supplementary material for: ﻿Evolutionary dynamics of the B chromosomes in the fish species Prochiloduslineatus Valenciennes, 1837 ﻿of the Paraná River Basin
Source: Comp Cytogenet. 2025 Jan 20;19:1–12. doi: 10.3897/compcytogen.19.135127 (PMC11773350; doi:10.3897/compcytogen.19.135127)
Supplement: Supplementary material 1 — Number of B chromosome in P.lineatus of Mogi Guaçu population [file comparative_cytogenetics-19-001_article-135127__-s001.docx]

**Suplementary files**

| **Suplementary table 1. Number of B chromosome in *P. lineatus*  of Mogi Guaçu population. A = acrocentric, M = metacentric, SM = submetacentric.** | | | | |
| --- | --- | --- | --- | --- |
| **Specimen** | **Number of Bs** | **A** | **M** | **SM** |
| 7212 | 2 | 0 | 2 | 0 |
| 7213 | 3 | 0 | 2 | 1 |
| 7214 | 3 | 0 | 2 | 1 |
| 7215 | 3 | 0 | 2 | 1 |
| 7216 | 2 | 1 | 1 | 0 |
| 7217 | 1 | 0 | 1 | 0 |
| 7218 | 3 | 0 | 3 | 0 |
| 7219 | 1 | 0 | 1 | 0 |
| 7220 | 1 | 0 | 1 | 0 |
| 7221 | 4 | 0 | 3 | 1 |
| 7222 | 2 | 0 | 2 | 0 |
| 7223 | 5 | 0 | 3 | 2 |
| 7226 | 2 | 0 | 2 | 0 |
| 7227 | 1 | 0 | 0 | 1 |
| 7230 | 4 | 0 | 3 | 1 |
| 7232 | 1 | 0 | 1 | 0 |
| 7233 | 4 | 0 | 3 | 1 |
| 7234 | 4 | 1 | 2 | 1 |
| 7235 | 1 | 0 | 1 | 0 |
| 7237 | 3 | 0 | 3 | 0 |
| 7238 | 2 | 0 | 1 | 1 |
| 7239 | 2 | 0 | 1 | 1 |
| 7240 | 2 | 0 | 2 | 0 |
| 7241 | 1 | 0 | 1 | 0 |
| 7242 | 4 | 0 | 3 | 1 |
| 7243 | 5 | 0 | 4 | 1 |
| 7244 | 3 | 0 | 3 | 0 |
| 7245 | 1 | 0 | 1 | 0 |
| 7248 | 1 | 0 | 1 | 0 |
| 7250 | 1 | 0 | 1 | 0 |
| 7251 | 3 | 0 | 2 | 1 |
| 7252 | 3 | 0 | 2 | 1 |
| **Total** | 78 | 2 | 60 | 16 |

| **Suplementary table 2. Number of B chromosome in *P. lineatus*  of Sapucaí population. A = acrocentric, M = metacentric, SM = submetacentric.** | | | | |
| --- | --- | --- | --- | --- |
| **Specimen** | **Number of Bs** | **A** | **M** | **SM** |
| 6740 | 3 | 0 | 2 | 1 |
| 6743 | 4 | 0 | 2 | 2 |
| 6744 | 2 | 0 | 2 | 0 |
| 6746 | 3 | 1 | 2 | 0 |
| 6747 | 4 | 0 | 4 | 0 |
| 6750 | 2 | 0 | 2 | 0 |
| 6751 | 2 | 0 | 2 | 0 |
| 6754 | 5 | 1 | 3 | 1 |
| 7189 | 1 | 0 | 1 | 0 |
| 7190 | 2 | 0 | 1 | 1 |
| 7191 | 3 | 2 | 1 | 0 |
| 7192 | 3 | 0 | 3 | 0 |
| 7195 | 2 | 0 | 2 | 0 |
| 7197 | 3 | 1 | 1 | 1 |
| 7199 | 3 | 1 | 2 | 0 |
| 7201 | 3 | 1 | 2 | 0 |
| 7203 | 4 | 0 | 4 | 0 |
| 7206 | 3 | 0 | 3 | 0 |
| 7207 | 1 | 0 | 1 | 0 |
| 7208 | 1 | 0 | 1 | 0 |
| 7209 | 2 | 0 | 2 | 0 |
| 7210 | 2 | 0 | 2 | 0 |
| 9250 | 3 | 1 | 2 | 0 |
| **Total** | 61 | 8 | 47 | 6 |

**Suplementary table 3. Number of B chromosome in *P. lineatus*  of Grande population. A = acrocentric, M = metacentric, SM = submetacentric.**

|  | | | | |
| --- | --- | --- | --- | --- |
| **Specimen** | **Number of Bs** | **A** | **M** | **SM** |
| 9257 | 5 | 0 | 3 | 2 |
| 9258 | 4 | 1 | 3 | 0 |
| 9259 | 4 | 2 | 2 | 0 |
| 9260 | 4 | 2 | 2 | 0 |
| 9262 | 4 | 1 | 3 | 0 |
| 9264 | 3 | 1 | 2 | 0 |
| 9265 | 2 | 0 | 2 | 0 |
| 9266 | 4 | 0 | 4 | 0 |
| 9267 | 2 | 0 | 2 | 0 |
| 9269 | 1 | 0 | 1 | 0 |
| 9270 | 2 | 0 | 1 | 1 |
| 9271 | 3 | 0 | 2 | 1 |
| 9272 | 5 | 1 | 2 | 2 |
| 9274 | 1 | 0 | 1 | 0 |
| 9275 | 2 | 0 | 2 | 0 |
| 9278 | 5 | 1 | 3 | 1 |
| 9279 | 2 | 0 | 1 | 1 |
| 9280 | 6 | 1 | 5 | 0 |
| 9281 | 6 | 1 | 4 | 1 |
| 9282 | 2 | 1 | 1 | 0 |
| **Total** | 67 | 12 | 46 | 9 |

**Suplementary table 4. Number of B chromosome in *P. lineatus*  of Batalha (Reginópolis) population. A = acrocentric, M = metacentric, SM = submetacentric.**

|  | | | | |
| --- | --- | --- | --- | --- |
| **Specimen** | **Number of Bs** | **A** | **M** | **SM** |
| 9215 | 2 | 0 | 2 | 0 |
| 9218 | 0 | 0 | 0 | 0 |
| 9235 | 3 | 0 | 3 | 0 |
| 9236 | 2 | 0 | 2 | 0 |
| 9292 | 2 | 0 | 2 | 0 |
| 9293 | 2 | 0 | 2 | 0 |
| 9294 | 2 | 0 | 2 | 0 |
| 9295 | 2 | 0 | 2 | 0 |
| 9296 | 2 | 0 | 2 | 0 |
| 9297 | 2 | 0 | 2 | 0 |
| 9298 | 3 | 0 | 3 | 0 |
| 9299 | 3 | 0 | 3 | 0 |
| 9300 | 2 | 0 | 2 | 0 |
| 9301 | 3 | 0 | 3 | 0 |
| 9302 | 1 | 0 | 1 | 0 |
| **Total** | 31 | 0 | 31 | 0 |

| **Suplementary table 5. Number of B chromosome in *P. lineatus*  of Batalha (Bauru) population. A = acrocentric, M = metacentric, SM = submetacentric.** | | | | |
| --- | --- | --- | --- | --- |
| **Specimen** | **Number of Bs** | **A** | **M** | **SM** |
| 9173 | 5 | 0 | 5 | 0 |
| 9174 | 6 | 0 | 6 | 0 |
| 9361 | 3 | 0 | 3 | 0 |
| 9363 | 3 | 0 | 3 | 0 |
| 9364 | 2 | 0 | 2 | 0 |
| 9365 | 0 | 0 | 0 | 0 |
| 9366 | 2 | 0 | 2 | 0 |
| 9367 | 3 | 0 | 3 | 0 |
| **Total** | 24 | 0 | 24 | 0 |

**Suplementary table 6. Number of B chromosome in *P. lineatus*  of Apa population. A = acrocentric, M = metacentric, SM = submetacentric.**

|  | | | | |
| --- | --- | --- | --- | --- |
| **Specimen** | **Number of Bs** | **A** | **M** | **SM** |
| C1 | 0 | 0 | 0 | 0 |
| C2 | 0 | 0 | 0 | 0 |
| C3 | 0 | 0 | 0 | 0 |
| C4 | 0 | 0 | 0 | 0 |
| C5 | 0 | 0 | 0 | 0 |
| C6 | 0 | 0 | 0 | 0 |
| C7 | 0 | 0 | 0 | 0 |
| C8 | 0 | 0 | 0 | 0 |
| C9 | 0 | 0 | 0 | 0 |
| C10 | 0 | 0 | 0 | 0 |
| C11 | 0 | 0 | 0 | 0 |
| C12 | 0 | 0 | 0 | 0 |
| C13 | 0 | 0 | 0 | 0 |
| C14 | 0 | 0 | 0 | 0 |
| C15 | 0 | 0 | 0 | 0 |
| C16 | 0 | 0 | 0 | 0 |
| C17 | 0 | 0 | 0 | 0 |
| C18 | 0 | 0 | 0 | 0 |
| C19 | 0 | 0 | 0 | 0 |
| C20 | 0 | 0 | 0 | 0 |
| Total | 0 | 0 | 0 | 0 |

**Suplementary table 7. Number of B chromosome in *P. lineatus*  of Paraná population. A = acrocentric, M = metacentric, SM = submetacentric.**

|  | | | | |
| --- | --- | --- | --- | --- |
| **Specimen** | **Number of Bs** | **A** | **M** | **SM** |
| 9643 | 1 | 0 | 1 | 0 |
| 9644 | 2 | 0 | 2 | 0 |
| 9645 | 2 | 0 | 2 | 0 |
| 9646 | 1 | 0 | 1 | 0 |
| 9647 | 2 | 0 | 2 | 0 |
| 9648 | 2 | 0 | 2 | 0 |
| 9649 | 1 | 1 | 0 | 0 |
| 9650 | 1 | 0 | 1 | 0 |
| 9651 | 0 | 0 | 0 | 0 |
| 9652 | 0 | 0 | 0 | 0 |
| 9653 | 1 | 0 | 1 | 0 |
| 9655 | 2 | 0 | 2 | 0 |
| 9656 | 1 | 0 | 1 | 0 |
| 9657 | 0 | 0 | 0 | 0 |
| 9658 | 1 | 0 | 1 | 0 |
| 9659 | 2 | 0 | 2 | 0 |
| 9660 | 3 | 0 | 3 | 0 |
| 9661 | 1 | 0 | 1 | 0 |
| 9662 | 0 | 0 | 0 | 0 |
| 9663 | 1 | 0 | 1 | 0 |
| 9664 | 1 | 0 | 1 | 0 |
| 9665 | 1 | 0 | 1 | 0 |
| 9666 | 2 | 0 | 2 | 0 |
| 9667 | 1 | 0 | 1 | 0 |
| 9668 | 2 | 0 | 2 | 0 |
| 9669 | 1 | 0 | 1 | 0 |
| 9670 | 1 | 0 | 1 | 0 |
| 9672 | 1 | 0 | 1 | 0 |
| 9673 | 1 | 0 | 1 | 0 |
| 9674 | 2 | 0 | 2 | 0 |
| **Total** | 37 | 0 | 36 | 0 |
